# Disparate miRNA expression in serum and plasma of patients with acute myocardial infarction: a systematic and paired comparative analysis

**DOI:** 10.1038/s41598-020-61507-z

**Published:** 2020-03-25

**Authors:** Ana Mompeón, Luis Ortega-Paz, Xavier Vidal-Gómez, Tiago Januario Costa, Daniel Pérez-Cremades, Sergio Garcia-Blas, Salvatore Brugaletta, Juan Sanchis, Manel Sabate, Susana Novella, Ana Paula Dantas, Carlos Hermenegildo

**Affiliations:** 1 Department of Physiology, Faculty of Medicine and Dentistry, University of Valencia, Valencia, Spain; 2 INCLIVA Biomedical Research Institute, Valencia, Spain; 3 Cardiology Division, Hospital Clínico Universitario de Valencia (HCUV), INCLIVA Biomedical Research Institute, Universidad de Valencia, Centro de Investigación Biomédica en Red de Enfermedades Cardiovasculares (CIBERCV), Valencia, Spain; 4 Institut d’Investigacions Biomèdiques August Pi i Sunyer (IDIBAPS), Barcelona, Spain; 5 Institut Clinic Cardiovascular (ICCV), Hospital Clinic i Provincial de Barcelona (HCPB), Barcelona, Spain

**Keywords:** miRNAs, Predictive markers

## Abstract

Despite the promising value of miRNAs in the diagnostic and prognostic of cardiovascular disease (CVD), recent meta-analyses did not support their potential. Methodological variances in studies may interfere with miRNA profile and affect their results. This study determines if the blood starting material is a source of variance in miRNA profile by performing a paired comparison in plasma and serum of the expression of primary miRNAs associated with CVD. Circulating miRNA yield was similar in both plasma and serum, although a significant increase was observed in patients with Non-ST-elevation myocardial infarction (NSTEMI) compared to control volunteers. When normalized by the expression of miR-484, different patterns of miRNA expression between serum and plasma. Although NSTEMI modified the expression of miR-1 and miR-208 in both serum and plasma, plasma displayed a higher variance than serum (Levene’s test p < 0.01). For miR-133a and miR-26a, differences were only detected in serum (p = 0.0240), and conversely, miR-499a showed differences only in plasma of NSTEMI (p = 0.001). Interestingly, miR-21 showed an opposite pattern of expression, being increased in serum (2^−ΔΔCt^: 5.7, p = 0.0221) and decreased in plasma (2^−ΔΔCt^: 0.5, p = 0.0107). Plasma and serum exhibit different patterns of circulating miRNA expression in NSTEMI and suggest that results from studies with different starting material could not be comparable.

## Introduction

MicroRNAs (miRNA) are highly conserved small noncoding RNAs that regulate post-transcriptional gene expression^1^. Through this mechanism, miRNAs regulate silencing of gene expression and can modify cell and tissue phenotype. Even though the existence of miRNAs and their mechanisms of action are not recent discoveries, the clinical implications of these micro molecules are relatively new and still a focus of debate. miRNAs can act intracellularly^1^ or be actively secreted by cells and contribute to intercellular or cell-tissue communication^1^. miRNAs are remarkably stable in human biofluids^2^, including plasma and serum, due to their packaging into membranous vesicles including exosomes, microvesicles, and apoptotic bodies, and to the association to RNA-binding proteins, such as the Argonaute family of lipoprotein complexes like high-density lipoprotein^3^. As a result, circulating miRNAs have emerged as powerful diagnostic or prognostic biomarkers in different pathophysiological conditions, including cardiovascular disease (CVD)^4^.

A large number of independent clinical studies and basic science in animal models have described distinct patterns of miRNA expression in CVD and associated risk factors, reinforcing the potential use of miRNA as molecular tools for the detection and prognosis of CVD^5,6^. Independent studies in patients with CVD show distinct circulating miRNA profile, among them the muscle- and heart-enriched miRNAs miR-1, miR-21, miR-133a, miR-208b, and miR-499a were consistently found elevated in the bloodstream of patients with acute myocardial infarction^6^. Besides, miR-26a, an enriched miRNA in the endothelium, is emerging as a crucial regulator of endothelial function and a biomarker of the progression of CVD^7^. Nevertheless, despite the initial excitement on the clinical value of miRNAs, recent meta-analyses were not able to provide enough evidence on their promising as a biomarker for CVD. Based on the current data, scientists could not establish that a specific miRNA profile works as a better method than the standardized biochemical and immunochemical assays currently used for diagnosis or prognosis of CVD^8,9^.

Differences in the time-point, heterogeneity in the population studied, or the presence of comorbidities are among factors that could diminish the power of meta-analysis. Also, besides inter-study variability, the field still needs to overcome several methodological challenges before introducing miRNA into the clinics. For instance, methods for miRNAs normalization and their interaction with circulating drugs remain unresolved^10^. Studies have shown that heparin administration and other medications during the acute cardiovascular event can alter circulating miRNA concentrations or their quantification^11^. Besides, differences in blood sampling may significantly interfere with the expression of nucleic acids, which could include miRNAs^12^. Different collecting tubes showed different concentrations of circulating miRNAs, and tubes with lithium-heparin were found unsuitable for miRNA quantification^12^. Furthermore, different analysis platforms used to quantify miRNA expression and to identify circulating miRNA profiles can profoundly influence the study outcome^10^.

In this study, we aimed to determine whether sample choice is a source of variation in the analysis of miRNA profile in coronary artery disease. We compared the expression of miRNAs mostly described in association with acute myocardial infarction (miR-1, miR-21, miR-26a, miR-133a, miR-208b, and miR-499a) in paired plasma and serum samples from patients Non-ST-elevation myocardial infarction (NSTEMI) relative to paired samples from control volunteers. By using a consistent paired protocol, we controlled other possible sources of variability including (1) population studied, as we collected samples from the same patients; (2) pre-analytical factors, as samples were obtained, processed and stored at the same time, (3) analytical factors, such as extraction protocol, cDNA synthesis and qPCR protocol; and (4) normalization procedure, as we used the same miRNA (miR-484) as endogenous control.

## Results

### Clinical baseline characteristics

A total of 74 patients with acute myocardial infarction without ST-elevation and who underwent primary PCI at HCPB cohort (n = 35) and HCUV (n = 39) were screened. Out of these, 66 were finally considered in the analysis as NSTEMI group, since only samples which had no hemolysis [(<0.03% hemolysis and ΔCq (miR-451 - miR-484) <5] and showed RNA quality proceeded to miRNA isolation and quantitation by qPCR. During the same period, a total of 20 samples from control volunteers were screened in the Valencian Biobanking Network and included in the analysis as a control group. Baseline clinical characteristics of the two groups are summarized in Table 1, and specific characteristics of patients at the time of inclusion are shown in Table 2. NSTEMI group showed on admission a higher rate of cardiovascular risk factors compared to control, including diabetes, hypertension, and hypercholesterolemia. No differences were found either in the body mass index and in the frequency distribution of samples by sex.

**Table 1:** Baseline Characteristics of NSTEMI Patients and Control.

	Control Group	NSTEMI Group	p Value
Age, mean± SD	64.1 ± 4.2	67.4 ± 11.0	0.0754
Sex, n (%)			
Male	15 (75)	49 (74)	0.9458
Female	5 (25)	17 (26)	
BMI, mean± SD	26.8 ± 3.2	27.5 ± 4.2	0.4944
Diabetes mellitus, n (%)	2 (10)	26 (39)	0.0148
Hypertension, n (%)	2 (10)	51 (77)	<0.0001
Hypercholesterolemia, n (%)	1 (5)	38 (58)	<0.0001
Family history of CAD, n (%)	0 (0)	7 (11)	0.1929

Data are shown as a percentage (%) of frequency or mean ± standard deviation (SD), as indicated. BMI - Body Mass Index; CAD - Coronary Artery Disease.

**Table 2:** Characteristics of NSTEMI patients at the time of inclusion.

Previous AMI, n (%)	14 (21)
Previous PCI, n (%)	14 (21)
Type of coronary lesion, N (%)	
LMC	3 (5)
LAD	47 (71)
Cx	30 (45)
RCA	34 (52)
Creatinine (mg/dl), median [IQR]	0.92 [0.8–1.3]
Hemoglobin (g/dl), median [IQR]	13.3 [11.6–14.4]
Platelets count, median [IQR]	217 [174–270]
Troponin peak (ng/ml), median [IQR]	71.6 [36.6–117.4]
Chronic medical treatment before admission, N (%)	
RAS Blocker	41 (62)
Beta Blocker	28 (42)
Statins	34 (52)
Calcium Channel Blocker	9 (14)
Diuretic	25 (38)
Anticoagulant	5 (8)
Hypoglycemic	18 (27)
Insulin	10 (15)

Data are shown as a percentage (%) of frequency or MEDIAN [IQR] as indicated. AMI - Acute Myocardial Infarction; PCI – Percutaneous Coronary Intervention; LMC – Left Main Coronary artery; LAD- Left Anterior Descending artery; Cx - Circumflex artery; RCA - Right Coronary Artery; RAS – Renin-Angiotensin System.

### Assessment of hemolysis

Rupture of red blood cells can release miRNA to serum or plasma and modify the analysis results^13,14^. Threfore, to exclude the variable “hemolysis” from our paired comparison, we determined the degree of hemolysis in serum and plasma samples and stablished a threshold to include samples in this study. After a first visual screening for pink/red discoloration (indicative of severe hemolyzed samples), hemolysis in the remaining serum and plasma samples was determined by two methods: 1) absorbance of hemoglobin content by spectrophotometry; and 2) the ratio of miR-451 to miR-484 (ΔCt: Ct miR-484 – Ct miR-451). As shown in Table 3, the average percentage of hemolysis of samples included in the study did not exceed 0.03%, a threshold accepted for miRNA research, reported not to interfere with circulating miRNA expression^13,14^. Moreover, the ratio of miR-451 (known to be enriched in erythrocytes and increased in samples with hemolysis) relative to miR-484, a stable miRNA in our samples (ΔCt) was similar in all groups (Table 3) and on average below 5, the cut-off established as indicator of possible contamination by erythrocyte miRNA contamination^13,14^.

**Table 3:** Markers of hemolysis in serum and plasma samples.

Sample	% Hemolysis (p = 0.8349)	Δ Ct (miR484-miR451) (p = 0.5824)
Control Serum	0.0065 ± 0.021	1.73 ± 0.48
NSTEMI Serum	0.0066 ± 0.022	1.92 ± 1.06
Control Plasma	0.0076 ± 0.023	1.64 ± 0.35
NSTEMi Plasma	0.0124 ± 0.024	1.44 ± 0.29

Data are shown as mean ± standard deviation (SD) as indicated. Factorial ANOVA analyzed the dependence of data on two independent variables (blood sampling and myocardial infarction) with Bonferroni’s post-test. p-values are expressed with each column label.

### miRNA enrichment and yield in serum and plasma

**Figure 1:**
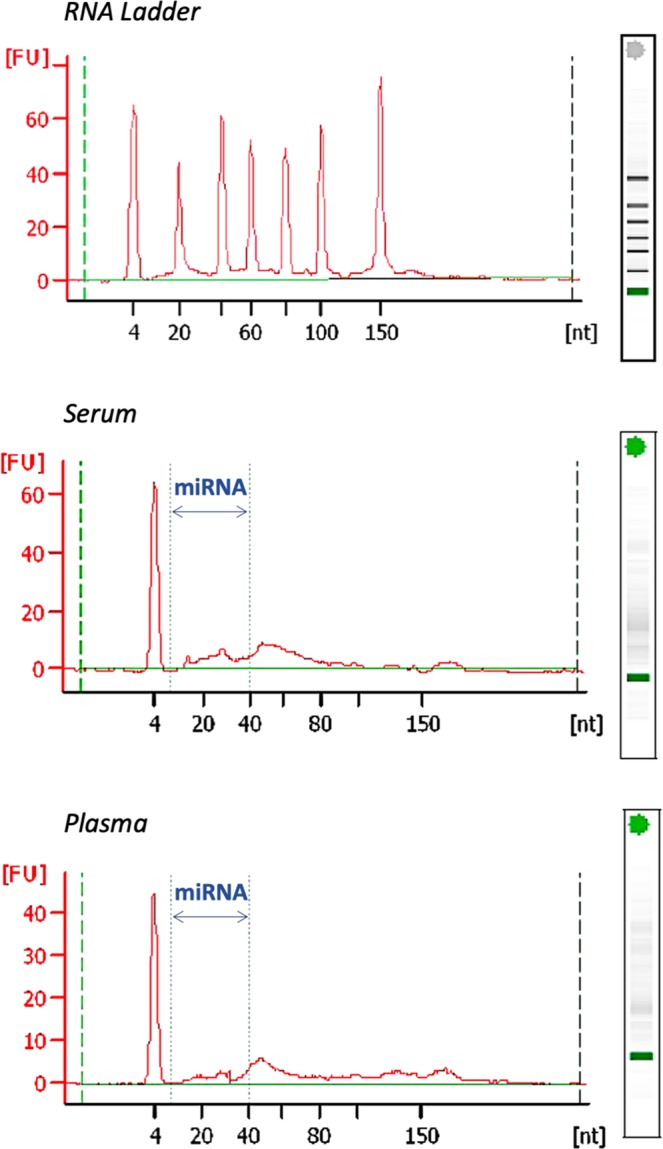
A representative composition of circulating small RNAs and miRNA fraction in serum and plasma, as indicated.

**Figure 2:**
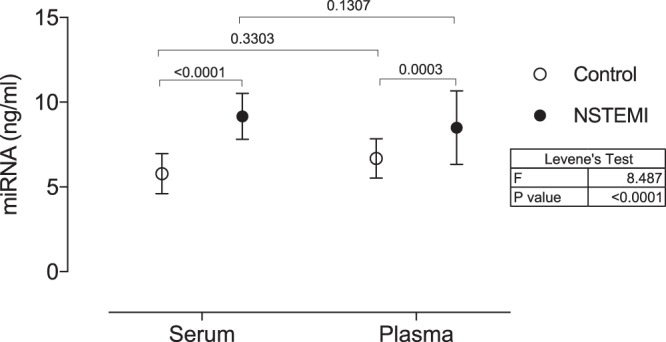
miRNA concentrations (ng/ml) in serum and plasma samples of NSTEMI patients in comparison to matched control determined by fluorometric quantitation. Data are shown as mean with 95% CI. P values of comparisons across the independent variables (blood sampling or myocardial infarction) are expressed on top of graphs. Variance across all groups (Levene’s test) is shown in the table next to the graph. Significance was considered when p < 0.05.

Samples were visualized by Agilent 2100 RNA bioanalyzer Pico Chip. Figure 1 shows the representative composition of circulating small RNAs (up to 200 nucleotides) and miRNA fraction [(miRNA/small RNA ratio (%)] in serum and plasma. There were no statistical differences when the mean and variances (SD) of miRNA fraction were compared between serum and plasma samples: [Mean (SD) - Serum: 33.7% (9.7); Plasma: 32.2% (5.2), p = 0.55]. Fluorimetric analysis of miRNA concentration by Qubit miRNA kit also shows that both serum and plasma yielded similar miRNA quantities when compared in control or NSTEMI groups. However, miRNA concentrations are significantly higher in both sample types of NSTEMI patients in comparison to matched control **(**Fig. 2).

### Choice of endogenous control to study miRNA expression in serum and plasma

**Figure 3:**
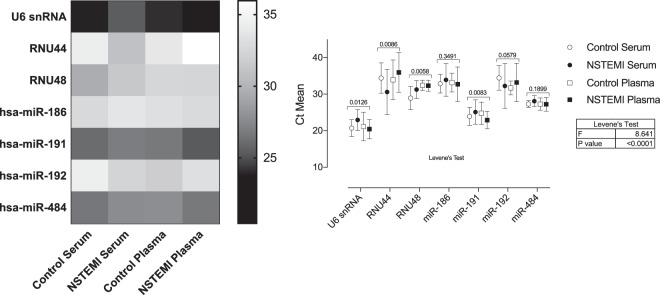
Heatmap and graphic representation of the mean expression (Ct) of 8 candidates for endogenous controls in serum and plasma of NSTEMI patients and control volunteers. Graphic data are shown as mean with 95% CI. P values of the analysis of variance (ANOVA) are expresses on top of each miRNA groupset. Significance was considered when p < 0.05.

In this study, we initially performed a detailed examination to establish a proper endogenous control suitable to be used in qPCR studies in both serum and plasma of NSTEMI patients and controls. The eight candidates studied were chosen based on the list of endogenous controls recommended by the manufacturer of TaqMan assays. For most control candidates, expression levels across sample types and experimental conditions (control or NSTEMI) were significantly different when means or standard deviation were tested **(**Fig. 3**)**. Four endogenous controls (U6 snRNA, RNU44, RNU48, and miR-191) exhibited significant differences when comparing the mean expression in plasma and serum from controls and NSTEMI. For the other three endogenous controls (miR-186 and miR-192), although they did not display differences between the means, they showed a high coefficient of variation (CV). When compared to miR-484, which showed the lowest CV (5.4%) and no significant difference between means, CV values of those three miRNAs were significantly higher [miR-186 (CV 10.8%, p = 0.0257); miR-192 (CV 13.5%, p = 0.0014)], according to Levene’s test. Values of miRNA of interest were, therefore, normalized to the expression of miR-484.

### miRNA expression in serum and plasma

In this study, we determined the limit of blank (LoB) in non-template controls from all miRNA studied before performing the expression analysis in serum and plasma samples to establish the maximun cycle threshod (Ct) value in which miRNA expression could be reliably detected [limit of quantitation (LoQ)]. For each miRNA, LoQ was determined as the Ct value that was 3.2 Ct units lower than the limit of blank (LoB). With these analyses, we observed Ct values ranging from 39–40 in non-template controls (data not shown); therefore, the LoQ in our samples was established as Ct of 35–36.5. Ct values with variance and number of samples included in the miRNA expression analysis is shown in Table 4.

**Table 4:** Values of cycle threshold (Ct) for miRNAs in serum and plasma samples.

		Ct Mean						Levene’s Test	
		Control			NSTEMI				
		Mean	SD	N	Mean	SD	N	F	P Value
miR-1	Serum	32.23	1.21	20	27.35	2.76	63	30.51	0.001
	Plasma	32.99	1.46	20	25.89	5.41	66		
miR-133a	Serum	31.09	2.28	20	28.11	2.67	64	22.10	0.001
	Plasma	30.57	3.09	20	27.38	5.65	66		
miR-208b	Serum	32.05	1.23	20	29.03	1.22	66	20.17	0.001
	Plasma	31.52	2.08	20	27.06	4.77	66		
miR-21	Serum	30.40	1.25	20	28.79	1.35	66	9.86	0.001
	Plasma	29.42	1.67	20	30.81	2.60	64		
miR-26a	Serum	30.59	1.04	20	32.76	2.15	62	29.04	0.001
	Plasma	30.25	2.14	20	31.40	5.61	60		
miR-499	Serum	31.24	1.39	20	29.96	3.05	66	14.98	0.001
	Plasma	32.91	1.75	20	29.09	5.03	64		

Data are shown as mean ± standard deviation (SD) of the number of samples as indicated. Levene’s test with Bonferroni correction calculated equality of variances across groups. p-values are expressed with each column label.

**Figure 4:**
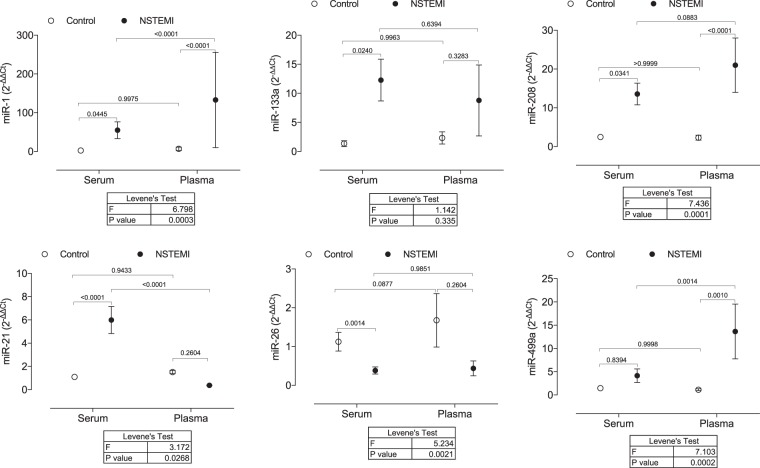
miRNA profile of most described miRNAs as potential biomarkers for CVD. miRNA expression of miR-1, miR-133a, miR-208b, miR-21, miR-26a, and miR-499a in serum and plasma samples of NSTEMI patients in comparison to matched control. Results are shown as mean with 95% CI. P values of comparisons across the independent variables (blood sampling or myocardial infarction) are expressed on top of graphs. Levene’s test with Bonferroni correction calculated equality of variances across groups. A level of p < 0.05 was used to assess statistical significance.

When considering only miRNA that passed LoQ threshold, a different pattern in miRNA expression was found in serum and plasma (Fig. 4). Although miR-1 and miR-208b were increased in both serum and plasma of NSTEMI patients, a significant difference in variance was found. When we compared variances between plasma and serum samples of NSTEMI, we observed that plasma samples displayed a higher CV than serum samples (Levene’s Test: miR-1, p = 0.0003; miR-208b, p = 0.0001). For other miRNAs, differences in expression were only detected in serum or plasma samples of NSTEMI. While miR-133a and miR26a levels were altered in the serum of NSTEMI, miR-499a expression was significantly increased only in plasma samples of NSTEMI. Curiously, miR-21 showed an opposite pattern of expression, being increased in serum (2^−∆∆Ct:^ 5.7, p = 0.0221) and decreased in plasma (2^−∆∆Ct:^ 0.5, p = 0.0107) of NSTEMI in comparison to control.

## Discussion

With this study, we draw attention to the importance of selecting the appropriate type of blood sample for specific miRNA determination and data comparison. Our data show that the type of specimen from the blood (serum or plasma) is a source of variation and influence the analysis of circulating miRNA in acute myocardial infarction. We evaluated the expression of primary miRNA associated with CVD and found a marked heterogeneity in the pattern of expression of all miRNA studied between serum and plasma. Although an increase of miR-1 and miR-208b was detected in both serum and plasma samples of NSTEMI, plasma samples displayed a marked variation (higher standard deviation). The expression of miR-133a and miR-26a was only modified in serum samples, and conversely, miR-499a showed differences only in plasma samples of NSTEMI. Surprisingly, miR-21 showed an opposite pattern of expression between samples, being increased in serum and decreased in plasma of NSTEMI. To the best of our knowledge, this is the first systematic and paired analysis on the effects of blood sampling on miRNA expression profile in association with CVD.

Analysis of miRNA profile may provide useful diagnostic and prognostic information, since changes in the miRNA expression may reflect the genetic and protein changes associated with the pathogenesis of many diseases^4^. There are different platforms available for determining variances of miRNA levels in human samples, all of which bring unique benefits and challenges in their use in mRNA profiling^15^. In this study, we opted to determine the expression of mature specific miRNAs by quantitative real-time PCR (qPCR) due to its high specificity and sensitivity in samples with low yield, as is the case of plasma and serum samples. qPCR is considered a gold standard technique in the detection and quantitation of gene expression and often used for validation of miRNA profile determined by other platforms. The primary and most apparent drawback of using the qPCR platform for the miRNA profile is its inability to identify new miRNAs. However, qPCR methodology can also be influenced by several factors that can hinder its specificity and sensitivity, thus increasing the variability of results and altering outcomes.

One of the challenges in analyzing miRNA expression in extracellular body fluid by qPCR is data normalization. To date, several small RNA species, such as U6 and snoRNAs have been used as endogenous control genes^16^. In our study, we investigated the expression of 8 candidates for endogenous controls. We selected as candidate reference genes the small nuclear RNA U6, two small nucleolar RNA (RNU44, RNU48), and four highly expressed miRNAs (miR-186, miR-191, miR-192, and miR-484) based on the list of endogenous controls recommended by the manufacturer of qPCR assays. Of these, miR-186, miR-192, and miR-484 could be cogitated as endogenous controls, if only considering that there was no significant difference among means. Nevertheless, when analyzing the variation of miRNA expression in the samples, we observed a significantly high coefficient of variation (~10%) in the expression of miR-186, miR-192, which may represent a difference of two PCR cycles, or 4-fold change, among samples in a giving group. As miR-484 showed low CV (5%) and no significant difference among means, the values of miRNA of interest were normalized to its expression. miR-484 has been proposed as a reference gene for studies in different types of cancer, determining miRNA expression as a biomarker for risk prediction^17,18^.

To determine the role of blood sampling on miRNA profile, we have selected six miRNAs that were the most described as potential biomarkers for CVD. miRNA choice was based on data presented in meta-analysis^8,9^ and systematic review^6^ of the literature, as well as in database for miRNA and disease association (HMDD, miR2Disease, Phenomir), using the following search terms: “Myocardial Infarction [MeSH]” OR “Coronary Artery Disease [MeSH]” OR “Acute Coronary Syndrome [MeSH]”. In the present study, we observed that samples from NSTEMI patients have a significantly higher concentration of total miRNA in the same starting volume of serum or plasma, which may be a consequence of increased miRNA release by apoptotic bodies resulting from acute cardiovascular event^19^. When equal amounts of total RNA were analyzed, a different pattern in miRNA expression was found in serum and plasma of NSTEMI. Inconsistencies were found either at the level of significance (miR-133a and miR-26a levels were significantly modified only in serum and miR-499a only in plasma of NSTEMI) or at the degree of variation (miR-1 and miR-208b showed a higher coefficient of variation in plasma samples). Curiously, miR-21 showed an opposite pattern of expression, being increased in serum and decreased in plasma of paired NSTEMI samples in comparison to control.

Because serum and plasma are derived from the same biofluid specimen, i.e., blood, the miRNA identified from either serum or plasma are erroneously compared and grouped in the literature. Serum and plasma are obtained from full blood, employing different biochemical processes in the laboratory. The serum is obtained from blood after fibrin clots formation during coagulation, whereas to obtain plasma, an anticoagulant is added before the removal of blood cells. These differences in the blood collecting procedures affect the coagulation cascade and may influence biochemical profiles in these matrices. The results obtained in miRNA determination from different biological fluids remain controversial, and so far, there is no standardization of methods for sampling, handling and preparation to ensure accurate quantitation and further establish a biomarker based on circulating miRNA. Differences in miRNA concentration from serum and plasma can be attributed to platelet contamination together with red and white blood cells^20,21^, hemolysis^14,20^, and the presence of qPCR inhibitors^11,22^. Previous studies on the exportation of miRNAs from cells proposed that plasma and serum exhibit some differences in their miRNA content^21,23^. In opposition, others have found little or no difference in specific extracellular miRNA quantification when comparing these biological fluids^24,25^.

Even though this study is not intended to serve as a recommendation for any particular sample type, in our analysis, serum demonstrated higher sensitivity and lower variability for most studied miRNA. The reasons behind the influence of blood sampling on miRNA expression are not clear, but the presence of a separator gel and additives in the tubes designed for sample processing may lead to a differential hemolytic contamination in serum and plasma that is independent of the extraction yields. In the present study, we observed no significant differences in the expression of miR-451, a miRNA enriched in red blood cells that can be released and modified in plasma and serum even by low degrees of hemolysis. In this regard, we can exclude this variable from the results observed. Similarly, a previous study has shown no differences in the expression of miR-451 in plasma compared to matched serum^21^.

Besides sample contamination by miRNA from red blood cells rupture, miRNA profile in serum and plasma may also be influenced by platelet content or activation. Activated platelets are known to release miRNAs incorporated into microparticles or the effector protein Argonaute 2 (Ago2)^26,27^. In serum, platelet-derived miRNAs may be released by activation during the coagulation process, and when it comes to plasma, there may be residual contamination even after careful serial centrifugation to deplete platelet content. A recent study has described considerable variability in miRNA expression in plasma depending on its platelet content (standard plasma vs. poor platelet plasma)^28^. Also, Wang *et al*.^21^ have shown variance in the expression of some platelet-enriched miRNAs in serum and plasma. The cycle threshold (Ct) for the expression of miR-233 and miR-191 was significantly lower in plasma than in serum (~1 PCR cycle, p < 0.05), representing an increase of approximately 2 fold in the expression of those miRNAs in plasma. Both miRNAs are highly expressed in the platelets and have been shown to be released by activated platelets^23^. In this study, we found that miR-191expression (a potential endogenous control) was markedly higher in serum from NSTEMI patients than matched NSTEMI plasma samples (p = 0.008). No differences in miR-191 expression were observed in serum and plasma from controls.

In this regard, caution with analyzes in samples potentially contaminated by platelets should be considered, especially in the acute myocardial infarction where they play an essential role in their pathophysiology. Platelets activated during acute coronary syndrome release miRNA that can contribute to the progression of the disease^29^, and therefore, differences in platelet contents by distinct pre-analytical plasma or serum process may interfere with the results of miRNA expression. The demonstration that miR-21 in this study showed an opposite pattern of expression increased in serum and decreased in plasma of paired NSTEMI samples in comparison to control highlights the importance of carefully standardizing sample specimens, pre analytics methods, and analysis in order compare results from different studies. Although highly expressed in cardiovascular cells, such as endothelial cells and cardiomyocytes, miR-21 is also expressed in the platelets. Increased circulating levels of miR-21 have been described in patients with cardiovascular disease and have been mostly associated with cardiovascular dysfunction. However, few studies have associated changes in miR-21 with platelet activation, since antiplatelet therapy significantly decreases its expression in the plasma^23^. In the present study, miR-21 showed an opposite pattern of expression in serum and plasma of NSTEMI. Although we cannot directly establish this correlation, it is plausible that increased platelet activation during blood serum separation may result in increased miR-21 expression in serum of NSTEMI patients, an effect that is lost in platelet-depleted plasma.

Beside the influence of blood cells on circulating miRNA expression, additives present in serum and plasma separation tubes could also interfere with miRNA quantitation. K2EDTA is an important chelator and could interfere with PCR reaction, by binding with magnesium chloride, an important source of magnesium ion for PCR, and influence primer annealing temperature, fidelity, specificity, and yield of PCR product^30,31^. Although different steps of RNA isolation usually result in the removal of most EDTA in the sample, traces of K2 could become a significant issue if the miRNA targets are present at low concentrations or when primers are relatively low in sensitivity and specificity. Besides, changes in astringency can be particularly critical in miRNA research due to their short sequence length and high sequence similarity among miRNA family members, sometimes as low as one nucleotide.

In conclusion, our study highlights the importance of proper and systematic standardization of sample collection when measuring circulating miRNAs and before establishing potential biomarkers based on circulating miRNA. We demonstrated that the choice of serum or plasma is essential to the experimental outcome and should be taking into account to avoid confounding results. Our work suggests that serum may be a more proper sample type in circulating miRNA studies in cardiovascular disease when considering its higher sensitivity and lower variability. On the other hand, platelet-poor plasma would be more recommendable when the circulating miRNA analyzed is higly expressed in platelets. We also propose that miR-484 could be used as a reference gene for circulating miRNA studies in cardiovascular diseases.

## Methods

### Study population and blood sampling

Consecutive patients were screened when admitted to the Hospital Clinic i Provincial de Barcelona (HCPB) and to the Hospital Clinic Universitari de Valencia (HCUV) for percutaneous coronary intervention (PCI) due to acute myocardial infarction without ST-elevation (NSTEMI, N = 66). Inclusion criteria were age ≤75 years, with more than one traditional cardiovascular risk factor. Exclusion criteria were previous stroke, thrombocytopenia, vasculitis, or any know immunological disorder, severe hepatic failure, uncontrolled hypertension (systolic or diastolic arterial pressure >180 mmHg or 120, respectively, despite medical therapy), limited life expectancy, e.g., neoplasms, inability to obtain informed consent and pregnancy. A group of volunteers (N = 20) without a history of acute myocardial infarction or chronic coronary disease and matched by age and sex was included as a control for comparative purposes. Samples and data from controls included in this study were provided by the BioBank IBSP-CV (PT13/0010/0064), integrated with the Spanish National Biobanks Network and the Valencian Biobanking Network. The baseline characteristics of the study population and clinical characteristics of NSTEMI patients at the time of inclusion are presented in Tables 1 and 2, respectively. Blood samples were collected prior to PCI into SST™ (for serum) and EDTA (for plasma) Vacutainer^®^ tubes and immediately sent (within 1 h) to the laboratory. Serum samples were allowed to cloth for 30 minutes at R.T., and plasma was incubated R.T. for the same amount of time. Samples were subjected to centrifugation at 1500 g for 15 min at 4 °C for plasma and serum separation. The supernatant was carefully transferred to a sterile tube and centrifuged again at 3000 g for 5 min at 4 °C in order to pellet any debris and insoluble components. Serum or plasma samples were aliquoted into 1.5-mL RNase-free Eppendorf DNA/RNA LoBind tubes (Eppendorf, Madrid, Spain) and immediately frozen at −80 °C until the day of experiments. All procedures in serum and plasma were run in parallel, i.e., they were performed at the same time and in the same way in both sample types. Written informed consent was obtained from all participants, and the studies were carried out under the approval of the Ethics Committee for Clinical Research (CEIC) of the HCPB (CEIC2013/8812) and HCUV (CEIC 2014/01/30), following the principles of the Declaration of Helsinki.

### Assessment of hemolysis

**Figure 5:**
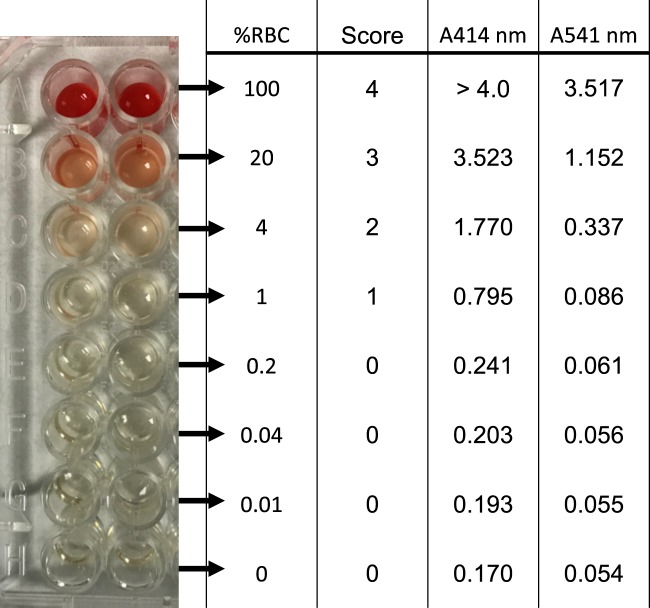
Representative curve for hemolysis assessment. A dilution series 1:5 of lysed red blood cells in plasma/serum was prepared. Hemolysis was assessed by visual score and spectrophotometry based on the optical density (OD) at 414 nm (absorbance peak of free hemoglobin) with additional peaks at 541 nm. Samples were classified as being hemolyzed if the OD at 414 nm exceeded 0.250 nm. For miRNA expression, samples were excluded when they displayed more than 0.03% of hemolysis.

Hemolysis reference was obtained by a 1:5 dilution series of sonicated whole blood from a healthy volunteer in non-hemolysed serum **(**Fig. 5**)**. A sample of commercial human serum (Sigma-Aldrich, St Louis, MI) was used as a reference of 0% hemolysis. The degree of hemolysis in serum and plasma samples was measured using three methods. Initially, we determined hemolysis with a simple visual inspection of samples for pink/red discoloration against a white background. Visible discoloration of each sample was scored from 0 (non-hemolyzed) to 4 (100% hemolyzed). Only samples with scores 0 and 1 were considered for the subsequent analysis. The second method determined the level of free hemoglobin in serum and plasma samples by spectral analysis with absorbance peaks at 414 and 541 nm. Samples were plotted against a curve of hemolysis reference, as described above, and expressed as a percentage (%) of hemolysis. Lastly, we determined the ratio of expression of red blood cell-enriched miRNA (miR-451) to the endogenous miRNA control (miR-484) by quantitative Real-Time PCR (qPCR), using TaqMan MicroRNA Assays (Applied Biosystems, Thermo Fisher, Waltham, MA). Samples were analyzed in duplicate.

### miRNA isolation and quantification

Total circulating RNA (tRNA) was isolated from serum and plasma using the miRCURY RNA Isolation Kit for Biofluids (Exiqon, Vedbaek, Denmark) following the manufacturer’s instructions. An aliquot of 400 μL of serum or plasma per sample was transferred to new RNAse-free microcentrifuge tubes for lysis and protein precipitation with the provided solutions. tRNA was precipitated with isopropanol and loaded onto a spin chromatography column provided by the kit. Following recommended washes, tRNA was eluted by adding 50 μL of RNAse-free water to the column and incubating for 1 min before centrifugation at 15000 g at room temperature. microRNA was assessed for quality and quantity with a chip for Small RNA with the Agilent 2100 Bioanalyzer (Agilent Technologies, Santa Clara, CA) and by Invitrogen™ Qubit™ 3.0 Fluorometer (Thermo Fisher, Waltham, MA) respectively. Equal amounts (50 ng) of miRNA were used for reverse transcription (RT) using the TaqMan miRNA Reverse Transcription Kit and for amplification by qPCR, using TaqMan MicroRNA Assays (Applied Biosystems, Thermo Fisher, Waltham, MA) of the selected miRNAs, and most used endogenous control **(**Table 5**)**. Each sample was analyzed in duplicate, and the expression was calculated according to the 2^−ΔΔCt^ method considering a pull of RNA from control samples (serum or plasma) as a reference. Relative quantification was performed using QuantStudio™ Software. The limit of quantitation (LoQ) for each miRNA studied was determined based on the principle of the minimum signal-to-noise ratio of 10, a typical constant used to define the minimum concentration at which an analyte can be reliably quantified^32^. Therefore, LoQ was calculated as the Cycle Threshold (Ct) value that is 3.2 Ct units lower than the limit of blank (LoB) (Ct value detected in non-template controls, containing the primer pair and master mix, without RNA sample). This difference provides a factor that is 10 times higher in LoQ (assuming a doubling miRNA product per cycle) and considering that Ct values are inversely proportional to miRNA expression. Samples with Ct higher than the established LoQ were considered as not expressing the analyzed miRNA.

**Table 5:** MicroRNA (miR) index for individual assays used.

Assay Name	Assay ID	Assay Target Sequence	miRBase ID (v21) or NCBI Name (for Controls)	miRBase Alias
U6 snRNA	001973	GUGCUCGCUUCGGCAGCACAUAUACUAAAAUUGGAACGAUACAGAGAAGAUUAGCAUGGCCCCUGCGCAAGGAUGACACGCAAAUUCGUGAAGCGUUCCAUAUUUU	U6 snRNA	NA
RNU44	001094	CCUGGAUGAUGAUAGCAAAUGCUGACUGAACAUGAAGGUCUUAAUUAGCUCUAACUGACU	RNU44	U44
RNU48	001006	GAUGACCCCAGGUAACUCUGAGUGUGUCGCUGAUGCCAUCACCGCAGCGCUCUGACC	RNU48	U48
miR-186	002285	CAAAGAAUUCUCCUUUUGGGCU	hsa-miR-186-5p	hsa-miR-186(17)
miR-191	002299	CAACGGAAUCCCAAAAGCAGCUG	hsa-miR-191-5p	hsa-miR-191(17)
miR-192	000491	CUGACCUAUGAAUUGACAGCC	hsa-miR-192-5p	hsa-miR-192(17)
miR-451	001141	AAACCGUUACCAUUACUGAGUU	hsa-miR-451a	hsa-miR-451(17)
miR-484	001821	UCAGGCUCAGUCCCCUCCCGAU	hsa-miR-484	NA
miR-1	002222	UGGAAUGUAAAGAAGUAUGUAU	hsa-miR-1-3p	hsa-miR-1(20)
miR-133a	002246	UUUGGUCCCCUUCAACCAGCUG	hsa-miR-133a-3p	hsa-miR-133a(19)
miR-208b	002290	AUAAGACGAACAAAAGGUUUGU	hsa-miR-208b-3p	hsa-miR-208b(19)
miR-21	000397	UAGCUUAUCAGACUGAUGUUGA	hsa-miR-21-5p	hsa-miR-21(17)
miR-26a	000405	UUCAAGUAAUCCAGGAUAGGCU	hsa-miR-26a-5p	hsa-miR-26a(17)
miR-499	002427	AACAUCACAGCAAGUCUGUGCU	hsa-miR-499a-3p	hsa-miR-499-3p(17)

Assay ID: Applied Biosystems Assay ID (6 digits), unique for each miRNA assay. Target Sequence: The mature miRNA target sequence of a miRNA assay. miRBase ID: List of valid miRBase gene ID or name given to a mature miRNA target of the miRNA assay. The information is derived from TaqMan miRBase Database v21(2015). 7. miRBase Alias: List of mature miRNA names (all species) that are or were associated with the target sequence and a complete list of current valid aliases (of all species). If a name was once associated with the mature miRNA target, the latest miRBase release version where the name was listed is shown in parenthesis.

### Statistical analysis

Sample size was calculated based on the model *n* = *2x(Zα* + *Z1-β) 2δ2*/*Δ2* to estimate the minimum number of samples required in each experimental subgroup NSTEMI vs control to obtain an alpha error of 5% (Zα) [two-tailed test because the results could be bidirectional], 95% power (Z1-β) to detect approximately double exchange rate transcriptional expression between groups (Δ) and standard deviation levels (δ) of 0.7^33^.

Continuous variables were expressed as mean ± standard deviation (SD) or median with interquartile range (IQR), according to their distribution. Mann-Whitney U test was used to compare the continuous variables, whereas the chi-square test or Fisher’s exact test was used to compare the categorical variables, as appropriate. Comparison of means across the two independent variables (blood sampling and myocardial infarction) was performed by factorial ANOVA when equal variances are assumed or Brown Forsythe for unequal variances followed by a posthoc analysis with Bonferroni correction for multiple comparisons. Results are shown as mean with 95% confidence interval (CI). Equality of variances (SD) across groups was calculated for each dependent variable by Levene’s test, with Bonferroni correction to test the homogeneity of the variance between all levels of comparison. We used an α level of 0.05 to assess statistical significance. Data were analyzed using SPSS for Windows™ version 23.0 software (SPSS Inc. Chicago, USA).

## Data Availability

A more detailed description of the study population, blood sampling, miRNA isolation, and analyses, please contact adantas@clinic.cat.
